# Vaginal Lactobacilli Reduce *Neisseria gonorrhoeae* Viability through Multiple Strategies: An *in Vitro* Study

**DOI:** 10.3389/fcimb.2017.00502

**Published:** 2017-12-06

**Authors:** Claudio Foschi, Melissa Salvo, Roberto Cevenini, Carola Parolin, Beatrice Vitali, Antonella Marangoni

**Affiliations:** ^1^Microbiology, Experimental, Diagnostic and Specialty Medicine - DIMES, University of Bologna, Bologna, Italy; ^2^Department of Pharmacy and Biotechnology, University of Bologna, Bologna, Italy

**Keywords:** *Lactobacillus*, gonococcus, probiotics, STIs, gonorrhea

## Abstract

The emergence and spread of antimicrobial resistance in *Neisseria gonorrhoeae* (GC) underline the need of “antibiotic-free” strategies for the control of gonorrhea. The aim of this study was to assess the anti-gonococcal activity of 14 vaginal *Lactobacillus* strains, belonging to different species (*L. crispatus, L. gasseri, L. vaginalis*), isolated from healthy pre-menopausal women. In particular, we performed “inhibition” experiments, evaluating the ability of both lactobacilli cells and culture supernatants in reducing GC viability, at two different contact times (7 and 60 min). First, we found that the acidic environment, associated to lactobacilli metabolism, is extremely effective in counteracting GC growth, in a pH- and time-dependent manner. Indeed, a complete abolishment of GC viability by lactobacilli supernatants was observed only for pH values < 4.0, even at short contact times. On the contrary, for higher pH values, no 100%-reduction of GC growth was reached at any contact time. Experiments with organic/inorganic acid solutions confirmed the strict correlation between the pH levels and the anti-gonococcal effect. In this context, the presence of lactate seemed to be crucial for the anti-gonococcal activity, especially for pH values in the range 4.4–5.3, indicating that the presence of H^+^ ions is necessary but not sufficient to kill gonococci. Moreover, experiments with buffered supernatants led to exclude a direct role in the GC killing by other bioactive molecules produced by lactobacilli. Second, we noticed that lactobacilli cells are able to reduce GC viability and to co-aggregate with gonococci. In this context, we demonstrated that released-surface components with biosurfactant properties, isolated from “highly-aggregating” lactobacilli, could affect GC viability. The antimicrobial potential of biosurfactants isolated from lactobacilli against pathogens has been largely investigated, but this is the first report about a possible use of these molecules in order to counteract GC infectivity. In conclusion, we identified specific *Lactobacillus* strains, mainly belonging to *L. crispatus* species, able to counteract GC viability through multiple mechanisms. These *L. crispatus* strains could represent a new potential probiotic strategy for the prevention of GC infections in women.

## Introduction

*Neisseria gonorrhoeae* (GC) is a Gram-negative oxidase-positive coccoid bacterium, responsible of gonorrhea, one of the most common bacterial sexually transmitted infection (STI), worldwide (WHO, 2012; ECDC, 2015).

In women, uro-genital GC infections are often asymptomatic, thus remaining unnoticed and untreated, and providing a significant reservoir for further transmission (Detels et al., 2011). If the gonococcal infections are not detected and/or appropriately treated, they can result in severe complications and sequelae such as pelvic inflammatory disease, infertility, ectopic pregnancy, first trimester abortion, and less frequently, disseminated infections (Morgan and Decker, 2016). Moreover, gonorrhea has also been reported to favor the transmission and acquisition of HIV infection (Johnson and Lewis, 2008; Sheung et al., 2008; Chun et al., 2013).

In the absence of a gonococcal vaccine, public health control of gonorrhea relies primarily on an effective, accessible, and affordable antimicrobial treatment (Unemo, 2015). Unfortunately, GC has developed resistance to all antimicrobials introduced for treatment of gonorrhea. The resistance to many antimicrobials, including penicillins, tetracyclines, and fluoroquinolones, has rapidly emerged within 1–2 decades and spread internationally (Unemo, 2015; Workowski and Bolan, 2015). In addition, since the early 2000s, documented cases of treatment failures with extended-spectrum cephalosporins have been reported worldwide (Ohnishi et al., 2011; Unemo et al., 2012; Fifer et al., 2016).

Considering all the aspects mentioned above, it is clear that, globally, gonorrhea causes significant morbidity and socioeconomic consequences and that a method to reduce the number of gonococcal infections without the antibiotic use would be beneficial for public health (WHO, 2012).

In this context, it has been shown that probiotic formulations based on *Lactobacillus* species can be useful in the prevention and treatment of several urinary and vaginal tract infections (Barrons and Tassone, 2008; Bolton et al., 2008; Spurbeck and Arvidson, 2011). The protective role of lactobacilli against uro-genital pathogens can be exerted through different mechanisms including the production of various antibacterial compounds, the competitive exclusion and the interaction with epithelial cell membrane (Kaewsrichan et al., 2006; Borges et al., 2014; Parolin et al., 2015; Nardini et al., 2016; Calonghi et al., 2017). The crucial role of lactobacilli in a normal vaginal microbiota is strengthened by the demonstration that in case of bacterial vaginosis, a clinical condition characterized by the depletion of lactobacilli, a higher risk of STI transmission and acquisition, including gonorrhea, is reported (Wiesenfeld et al., 2003; Abbai et al., 2016).

The *in vitro* interaction between GC and lactobacilli has been previously investigated by several authors, and some of the mechanisms that lactobacilli use to prevent GC infection and to counteract GC viability, are known. Many studies have shown the ability of different strains of lactobacilli to reduce GC adhesion to epithelial cells (Spurbeck and Arvidson, 2008, 2010; Vielfort et al., 2008). Moreover, the production of bacteriocin-like substances and acidic compounds can inhibit GC viability, as previously demonstrated (Zheng et al., 1994; Graver and Wade, 2011; Breshears et al., 2015; Ruíz et al., 2015). Nevertheless, only a few data about GC and lactobacilli interaction have been conducted with *Lactobacillus* strains isolated from the vaginal niche and many aspects remain to be elucidated (Vielfort et al., 2008; Graver and Wade, 2011; Breshears et al., 2015; Ruíz et al., 2015).

For that reason, the aim of this study was to assess the *in-vitro* capacity of a wide panel of vaginal *Lactobacillus* strains, belonging to different species, to interfere with GC viability.

For these strains the inhibitory activity against other STI agents, such as *Chlamydia trachomatis* and HIV, has already been demonstrated (Nardini et al., 2016; Ñahui Palomino et al., 2017).

In particular, here, we performed “inhibition” experiments against GC with two lactobacilli fractions: culture supernatants were explored in order to evaluate the anti-GC activity of secreted bio-molecules, whereas cell pellets were tested to assess the capacity of surface components to reduce GC viability.

A major potential application of this study relies on the identification of active *Lactobacillus* strains to propose as probiotics for the prophylaxis of GC infections adversely affecting women's health.

## Materials and methods

### *Lactobacillus* strains, culture conditions and preparation of fractions

All the 14 *Lactobacillus* strains included in this study were previously isolated from vaginal swabs of healthy premenopausal Caucasian women (Parolin et al., 2015). The isolation technique, the species identification and the functional characterization of these strains are described elsewhere (Parolin et al., 2015; Foschi et al., 2017; Siroli et al., 2017).

In particular, 7 strains of *L. crispatus* (BC1, BC3-BC8), 5 strains of *L. gasseri* (BC9, BC10, BC12-BC14), and 2 strains of *L. vaginalis* (BC16, BC17) were employed.

Lactobacilli were cultured in de Man, Rogosa, and Sharpe (MRS) broth (Becton Dickinson and Company, Sparks, MD, USA) supplemented with 0.05% L-cysteine, incubated at 37°C. Anaerobic conditions were achieved by using anaerobic jars supplemented with GazPack EZ (Becton Dickinson and Company).

The turbidity of 18-h lactobacilli cultures was measured by a McFarland (McF) densitometer (DEN 1-B, BioScientifica, Italy), considering that a turbidity of 0.5 McF corresponds approximately to a cell concentration of 1.5 × 10^8^ colony forming unit (CFU)/mL. Lactobacilli cultures were adjusted to 1.6 McF with sterile saline (cell concentration: 5 × 10^8^ CFU/mL) and centrifuged at 5,000 × g for 10 min at 4°C. Supernatants were filtered through a 0.22 μm membrane filter to obtain stock cell free supernatants (CFS) and used for “inhibition” experiments. The pH values of CFS were measured by a pH Meter, after appropriate calibration (Table 1). Cell pellets were washed and re-suspended in phosphate-buffered saline (PBS) to obtain stock suspensions of 5 × 10^8^ CFU/mL for “inhibition” experiments.

**Table 1:** Correlation between pH values and the effect of lactobacilli CFS on GC viability.

**pH value of supernatant**	***Lactobacillus* strains**	**Anti-GC effect at 7 min**	**Anti-GC effect at 60 min**
≥4.5	*L. gasseri* BC10 *L. vaginalis* BC16	5% reduction of GC viability	70–90% reduction of GC viability
4–4.5	*L. crispatus* BC3 *L. gasseri* BC9, BC12, BC14 *L. vaginalis* BC17	70–99% reduction of GC viability	100% reduction of GC viability
<4	*L. crispatus* BC1, BC4–BC8 *L. gasseri* BC13	100% reduction of GC viability	100% reduction of GC viability

### *Neisseria gonorrhoeae* strain and culture conditions

*Neisseria gonorrhoeae* MS11 (ATCC BAA-1833) (Schoolnik et al., 1984) was grown at 37°C in a humidified 5% CO_2_ environment on Thayer Martin-VCNT (VCNT = vancomycin, colistin methane sulfonate, nystatine, and trimethoprim) agar plates (MEUS SRL, Piove di Sacco, Padova, Italy). Piliation and Opa phenotype were distinguished by colony morphology under a stereomicroscope (Swanson et al., 1971; Swanson, 1978; Bergström et al., 1986) and only piliated Opa-negative variants were used.

To exclude a significant reduction of GC viability due to a spontaneous autolysis, the survival rate of gonococci in control conditions (e.g., PBS, MRS medium) was checked prior to each experiment, proving to be comprised between 75 and 85%.

### “Inhibition” experiments

“Inhibition” experiments were carried out incubating 1 × 10^8^ lactobacilli cells or the corresponding supernatants with the same amounts of gonococcal cells (ratio gonococci: lactobacilli = 1), in order to evaluate a direct effect of lactobacilli fractions on GC viability. In details, 1 × 10^8^ gonococcal cells (200 μL of a PBS stock solution with a concentration of 5 × 10^8^ CFU/mL) were pelleted in an eppendorf tube and the supernatant was discarded. Afterward, 200 μL of the stock suspensions of lactobacilli cells or CFS were added and the mixes were incubated for 7 or 60 min at 37°C in 5% CO_2_ atmosphere. At the end of the incubation time, the tubes were centrifuged at 20,000 × g for 10 min. Pellets were re-suspended in PBS, serially-diluted (1:50, 1:100, 1:200, 1:500), and 10 μL of the diluted suspensions were seeded on Thayer-Martin agar plates. After 48 h of incubation (37°C, 5% CO_2_), gonococcal colonies were visually counted and expressed in number of gonococci/mL. Each experiment was conducted in duplicate and a control tube (1 × 10^8^ gonococcal cells incubated with 200 μL of PBS or MRS, without lactobacilli fractions) was always included.

Results were expressed as the percentage of the average number of gonococci/mL. The gonococcal growth in the presence of lactobacilli fractions was compared with the corresponding control (gonococcal growth in the absence of lactobacilli fractions), taken as 100%.

Finally, in order to verify the viability of lactobacilli cells after the interaction with gonococci, in case of cell pellets experiments, 10 μL of the same dilutions were seeded in parallel on MRS agar plates and incubated at 37°C in anaerobic conditions. After 48 h, lactobacilli colonies were numbered and compared with the growth of lactobacilli incubated without gonococci.

### “Inhibition” experiments with lactic acid and hydrochloric acid

Solutions of lactic acid and hydrochloric acid at a concentration of 10 mM in 150 mM NaCl and buffered to different pH values (3.4, 3.7, 4.0, 4.4, 4.7, 5.0, 5.3) with NaOH 1M were used for “inhibition” experiments against GC, as described above. One hundred and fifty millimolars of NaCl solution (pH 5.8) was used as control. Similarly, gonococcal inhibition experiments were performed with MRS broth buffered to different pH values (3.4, 3.7, 4.0, 4.4, 4.7, 5.0, 5.3) with lactic acid and hydrochloric acid solutions (molar range of acids: 5–50 mM), using MRS broth (pH: 6.0) as control.

### “Inhibition” experiments with buffered CFS

The supernatants of highly active *Lactobacillus* strains (BC1, BC4, BC5, BC6, BC7, BC8, BC13) were buffered to pH 6.0 with NaOH 1M and “inhibition” experiments were conducted in the same conditions described above.

### Methanol and proteinase K treatment of lactobacilli

*L. crispatus* BC1 and BC3 suspensions (5 × 10^8^ lactobacilli CFU/mL) were divided into two 200 μL-aliquots. Aliquots were treated with 500 μL of ice-cold methanol for 10 min and then centrifuged at 14,000 × g for 3 min to pellet the bacteria. The supernatant was removed and replaced with 200 μl of sterile saline (aliquot 1, methanol treated) or 160 μl of saline and 40 μl proteinase K (20 mg/ml) (aliquot 2, proteinase K treated). After an incubation for 2 h at 37°C, aliquots were boiled for 3 min and then centrifuged at 14,000 × g for 3 min to pellet the bacteria. The supernatants were removed and lactobacilli were directly re-suspended in 200 μl of a suspension of 5 × 10^8^ gonococci/mL, then “inhibition” experiments were carried out as previously described. Microscopic visualization of Gram-stained samples was performed to evaluate the effect of methanol and proteinase K on lactobacilli.

### Isolation of released surface components (RSC) and biosurfactant activity assessment

Released surface-associated components were isolated from BC1 and BC10 lactobacilli strains, following previous published protocol with slight modifications (Spurbeck and Arvidson, 2010; Sambanthamoorthy et al., 2014). Considering the cell pellet activity against GC, BC1 and BC10 lactobacilli were chosen as examples of a highly active and a non-active strain, respectively.

Briefly, a 1-liter culture of lactobacilli was grown for ~48 h in MRS broth to a turbidity of 8–10 McF, and the bacteria were harvested by centrifugation (10,000 × g for 10 min at 4°C). The bacterial cell pellet was washed twice with sterile water and then re-suspended in 100 mL of PBS. This suspension was gently stirred for 2 h at room temperature to release cell-bound bio-molecules and then centrifuged for 20 min at 3,000 × g. The supernatant was filtered through a 0.22 μm filter, to remove the remaining bacteria. This cell-free preparation was concentrated to ~10 ml by Amicon ultraconcentration using a 10,000-molecular-weight-cutoff filter membrane. The pH values of RSC were measured by a pH Meter, after appropriate calibration.

The biosurfactant activity of the RSC was evaluated by performing an oil spreading assay, as described elsewhere (Sambanthamoorthy et al., 2014). Briefly, 50 mL of demineralized water were poured into a 150 mm Petri dish and 20 μL of motor oil were added to the surface of the water. Ten microliters of RSC from either *L. crispatus* BC1 and *L. gasseri* BC10 were then added to the surface of the oil. Water was used as a negative control. The diameters of clear zones of triplicate assays from the same sample were determined.

RSC were finally employed in “inhibition” experiments, as previously described. Serial dilutions of BC1 and BC10 RSC were tested (1:1, 1:2, 1:4), in order to verify a dose-response effect. Gonococcal cells (1 × 10^8^) incubated in PBS were used as control.

### Co-aggregation experiments

Lactobacilli were screened for their capacity to interact and aggregate with piliated gonococci: 1 × 10^8^ gonococcal cells and 1 × 10^8^ lactobacilli CFU were mixed together in PBS in a final volume of 200 μl and incubated for 7 or 60 min at 37°C. Afterwards, an aliquot of the suspension was fixed on a microscope slide and Gram stained, in order to evaluate the bacterial spatial arrangement.

To strengthen the hypothesis of a specific interaction between lactobacilli and gonococci, a “highly aggregating” *Lactobacillus* strain (*L. crispatus* BC1) was used as a model for additional experiments. In particular, after 60 min contact time between BC1 and GC and prior to the Gram-staining, the bacterial pellet was subjected to 2 times PBS washing and low speed-centrifugation (1,200 × g for 10 min), to remove lactobacilli not specifically bound to gonococcal cells.

### Statistical analysis

All statistical analysis were performed by using GraphPad Prism software (GraphPad Prism version 5.02 for Windows, GraphPad Software, San Diego California USA, www.graphpad.com). Statistical analysis for “inhibition” assays were performed by using one-way analysis of variance (ANOVA) test, followed by Dunnett's Multiple Comparison test. Differences in the anti-GC activity between lactic acid and hydrochloric acid were searched by means of a paired *t*-test. Results were expressed as mean ± Standard Error of the Mean (SEM). Statistical significance was determined at ^*^P < 0.05, ^**^P < 0.01, and ^***^P < 0.0001.

## Results

### “Inhibition” experiments: effects of lactobacilli CFS on GC viability

**Figure 1:**
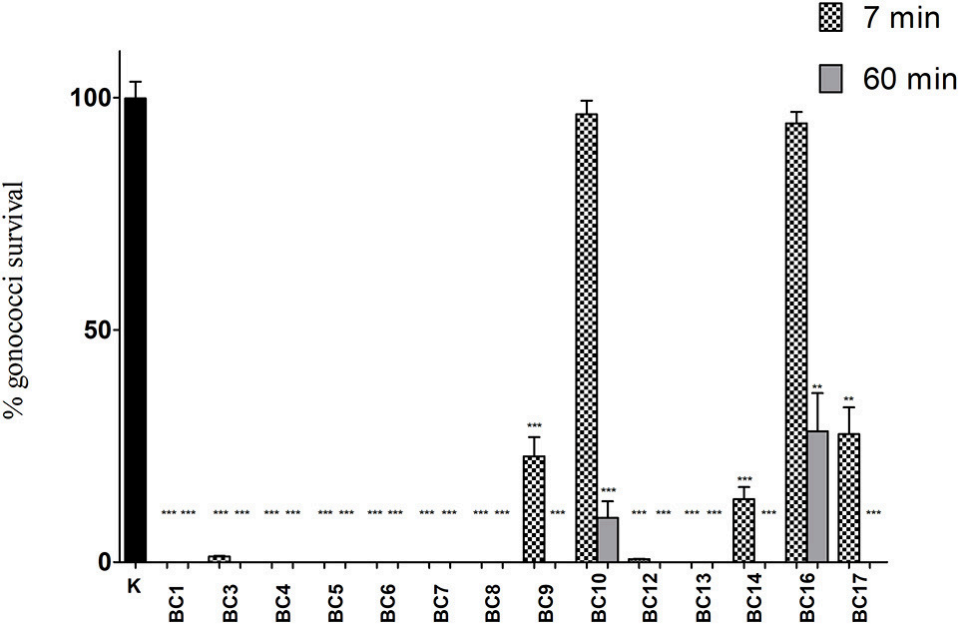
“Inhibition” experiments with lactobacilli CFS. Experiments were performed with a volume of supernatants corresponding to 1 × 10^8^ CFU of lactobacilli added to 1 × 10^8^ gonococcal cells at two different time points: 7 min (dotted bars) and 60 min (gray bars). GC viability was evaluated as number of gonococci/mL and expressed as % of gonococci survival. The results were expressed in percentage compared to control (K) (1 × 10^8^ gonococcal cells incubated in MRS), taken as 100% (black bar). Bars represent mean values, whereas error bars represent SEM. Statistical significance was determined at ^**^P < 0.01 and ^***^P < 0.0001.

In order to explore the anti-gonococcal activity of bio-molecules secreted by vaginal *Lactobacillus* strains, “inhibition” experiments were carried out with lactobacilli culture supernatants. The results obtained with CFS are shown in Figure 1. *L. crispatus* BC1, BC4, BC5, BC6, BC7, BC8 and *L. gasseri* BC13 CFS were able to completely abolish GC viability both at 7 and 60 min contact times. *L. crispatus* BC3, *L. gasseri* BC9, BC12, BC14, and *L. vaginalis* BC17 caused a 100%-reduction of GC viability only after 60 min, whereas *L. gasseri* BC10 and *L. vaginalis* BC16 were unable to fully prevent GC growth at both contact times, although a significant reduction was observed at 60 min. Globally, *L. crispatus* species showed the best antimicrobial profile, considering that all the lactobacilli supernatants of that species reduced GC viability, in a highly significant manner, both at 7 and 60 min.

As shown in Table 1, the effect of lactobacilli supernatants on GC viability was strictly related to the pH values and time of contact. Indeed, in case of pH values < 4.0 lactobacilli supernatants completely abolished GC growth at both contact times, whereas for pH values ranging from 4.0 to 4.4, GC was totally killed only at 60 min. Finally, when supernatant pH values were ≥ 4.5, no complete reduction of GC viability was observed at any time of contact.

### GC inhibition by lactic acid and hydrochloric acid

On the basis of the results of “inhibition” experiments with lactobacilli CFS, the same assays were carried out with lactic acid and hydrochloric acid solutions, buffered at different pH values, in order to evaluate the effect of organic/inorganic acids on GC viability. The concentration of acids (10 mM) was chosen because corresponding to the mean titer of lactate found in *Lactobacillus* CFS, as reported elsewhere (Nardini et al., 2016).

**Figure 2:**
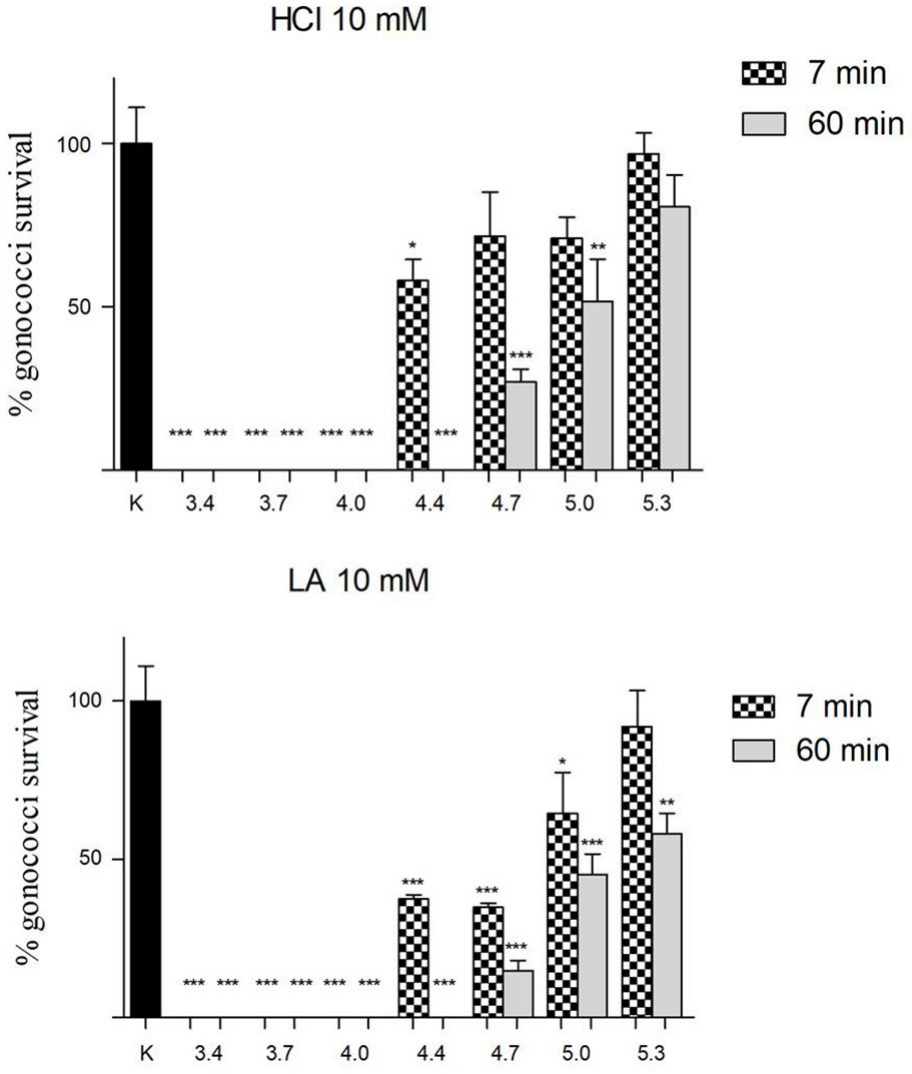
Effect of lactic and hydrochloric acid on GC viability. Solutions of hydrochloric acid (HCl) and lactic acid (LA) at a concentration of 10 mM, buffered to different pH values (3.4, 3.7, 4.0, 4.4, 4.7, 5.0, 5.3), were used for “inhibition” experiments against GC at two different time points (7 and 60 min; dotted and gray bars, respectively). GC viability was evaluated as number of gonococci/mL and expressed as % of gonococci survival. The results were expressed in percentage compared with control (K) (1 × 10^8^ gonococcal cells incubated in 150 mM NaCl solution, pH 5.8), taken as 100% (black bar). Bars represent mean values, whereas error bars represent SEM. Statistical significance was determined at ^*^P < 0.05, ^**^P < 0.01, and ^***^P < 0.0001.

As shown in Figure 2, for pH values ≤ 4.0, a complete abolishment of GC viability was noticed both for hydrochloric acid and for lactic acid solutions. Differently, in case of pH values in the range 4.4–5.3, lactic acid showed a higher anti-GC activity compared to hydrochloric acid, both at 7 and 60 min contact time. Globally, considering all the pH values and both the contact times, lactic acid was significantly more active than hydrochloric acid in reducing GC viability (*P* = 0.003).

Comparable results were obtained when “inhibition” experiments were carried out with MRS broth buffered to different pH values with lactic or hydrochloric acid (Figure S1).

### Experiments with buffered supernatants

To verify the central role of the acidic environment in counteracting GC viability and to exclude the involvement of other bioactive substances (e.g., H_2_O_2_) in anti-gonococcus activity, we performed “inhibition” experiments with the CSF of *Lactobacillus* active strains, buffered to pH 6.0.

Compared to controls, the lactobacilli supernatants buffered at pH values of 6.0 led to a not-significant reduction of GC viability, both at 7 and 60 min contact times (Figure S2).

### “Inhibition” experiments: effects of lactobacilli cells on GC viability

**Figure 3:**
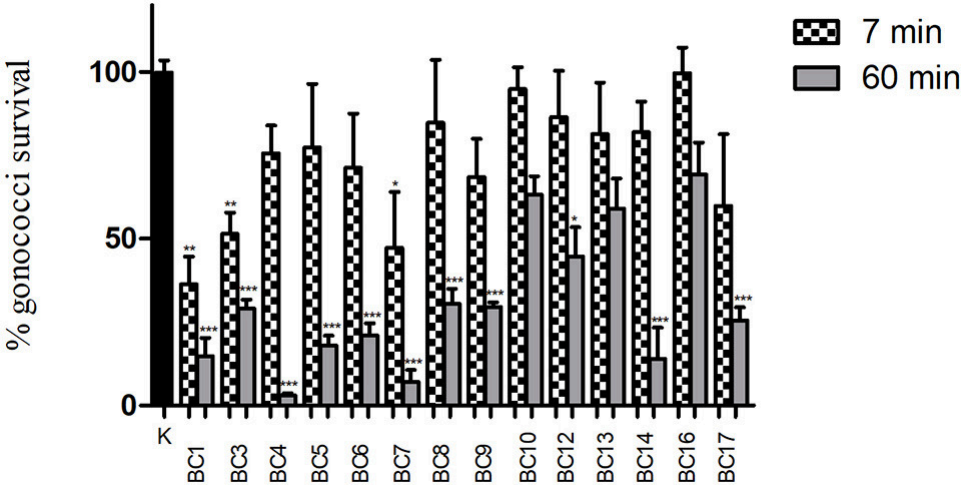
“Inhibition” experiments with lactobacilli cells. Experiments were performed with 1 × 10^8^ CFU of lactobacilli incubated with 1 × 10^8^ gonococcal cells at two different time points: 7 min (dotted bars) and 60 min (gray bars). GC viability was evaluated as number of gonococci/mL and expressed as % of gonococci survival. The results were expressed in percentage compared to control (K) (1 × 10^8^ gonococcal cells incubated in PBS, without lactobacilli), taken as 100% (black bar). Bars represent mean values, whereas error bars represent SEM. Statistical significance was determined at ^*^P < 0.05, ^**^P < 0.01, and ^***^P < 0.0001.

The capacity of lactobacilli surface components to reduce GC viability was investigated by means of “inhibition” experiments with 1 × 10^8^ lactobacilli. The results of “inhibition” experiments with lactobacilli are shown in Figure 3. Globally, cell pellets were far less effective compared to the corresponding supernatants, in particular at short contact times (7 min). In fact, no cell pellet was able to completely abolish GC viability at any time point. *L. crispatus* BC1, BC3 and BC7 were the only strains able to significantly reduce GC growth at 7 min, whereas all the lactobacilli, except for *L. gasseri* BC10 and BC13 and *L. vaginalis* BC16, were effective at 60 min, although at various levels. Overall, *L. crispatus* species showed the best activity against GC, in agreement with the results obtained with lactobacilli CFS. Lactobacilli cell viability was completely retained after the interaction with GC cells, at both contact times (data not shown).

### Methanol and proteinase K treatment of lactobacilli

“Inhibition” experiments were also carried out with lactobacilli cells treated with methanol and proteinase K prior to the interaction with gonococci. These assays were performed to evaluate if lactobacilli surface components were involved in GC inhibition and, finally, to determine the chemical nature of such components. On the basis of the anti-gonococcus activity of cell pellets, *L. crispatus* BC1 and BC3 were chosen as examples of highly active strains. After methanol and proteinase K treatment of BC1 and BC3 cells, the lactobacilli completely lost their anti-gonococcal activity, indicating that lactobacilli lipidic/proteic surface components are involved in the inhibition and that viable lactobacilli are needed for the GC killing (Figure S3).

Microscopic visualization of Gram-stained samples confirmed that methanol and proteinase K treatment left the lactobacilli intact (data not shown).

### Experiments with RSC

In order to evaluate the role of released surface-associated components in the inhibition of GC showed by cellular fraction, RSC were isolated from BC1 and BC10 lactobacilli strains. Considering their activity against GC, these two strains were chosen as examples of a highly active and a non-active strain, respectively.

*L. crispatus* BC1 RSC showed a pH value of 5.5 and possessed a high biosurfactant activity, evaluated by the oil spreading assay (diameter of the clear zone on the oil-water surface: 14 ± 0.5 mm), whereas BC10 RSC were characterized by a pH value of 6.4 and by a lower biosurfactant activity (diameter of the clear zone on the oil-water surface: 8 ± 0.1 mm).

**Figure 4:**
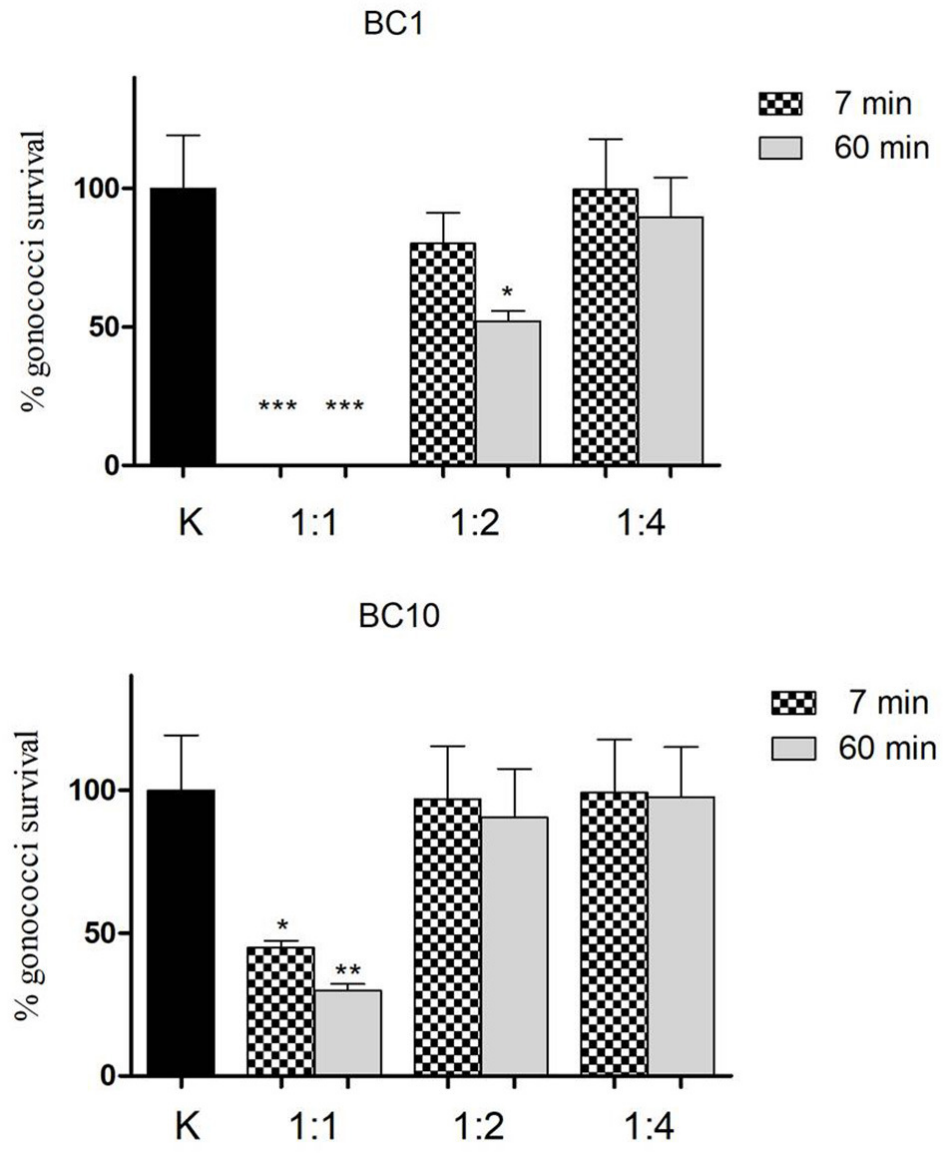
Effect of lactobacilli RSC on GC viability. “Inhibition” experiments against GC were performed with released surface components isolated from *L. crispatus* BC1 (BC1) and *L. gasseri* BC10 (BC10). Three different dilutions of RSC were tested (1:1, 1:2, and 1:4) at two different time points (7 min-dotted bars and 60 min-gray bars). GC viability was evaluated as number of gonococci/mL and expressed as % of gonococci survival. The results were expressed in percentage compared to control (K) (1 × 10^8^ gonococcal cells incubated in PBS), taken as 100% (black bar). Bars represent mean values, whereas error bars represent SEM. Statistical significance was determined at ^*^P < 0.05, ^**^P < 0.01, and ^***^P < 0.0001.

The effect of lactobacilli RSC on GC viability is shown in details in Figure 4. BC1-derived RSC were able to abolish completely GC viability both at short (7 min) and longer contact times (60 min), when tested as a whole. When diluted 1:2, the RSC retained a partial activity against GC (15%-reduction of GC viability at 7 min and 50%-reduction after 60 min), whereas in case of 1:4 dilution, the GC-killing activity was lost.

On the other hand, BC10 RSC were less effective compared to BC1 in reducing GC viability: after 7 min, a 50%-reduction of GC viability was found, whereas at 60 min contact time, the reduction reached the 70%. When diluted (both 1:2 and 1:4) BC10 RSC completely lost their activity against GC, at both contact times.

### Lactobacilli interaction with piliated GC

The capacity of lactobacilli to aggregate with piliated gonococci was screened, in order to evaluate if the killing effect displayed by lactobacilli cells could be associated to the ability to physically interact with gonococci.

**Figure 5:**
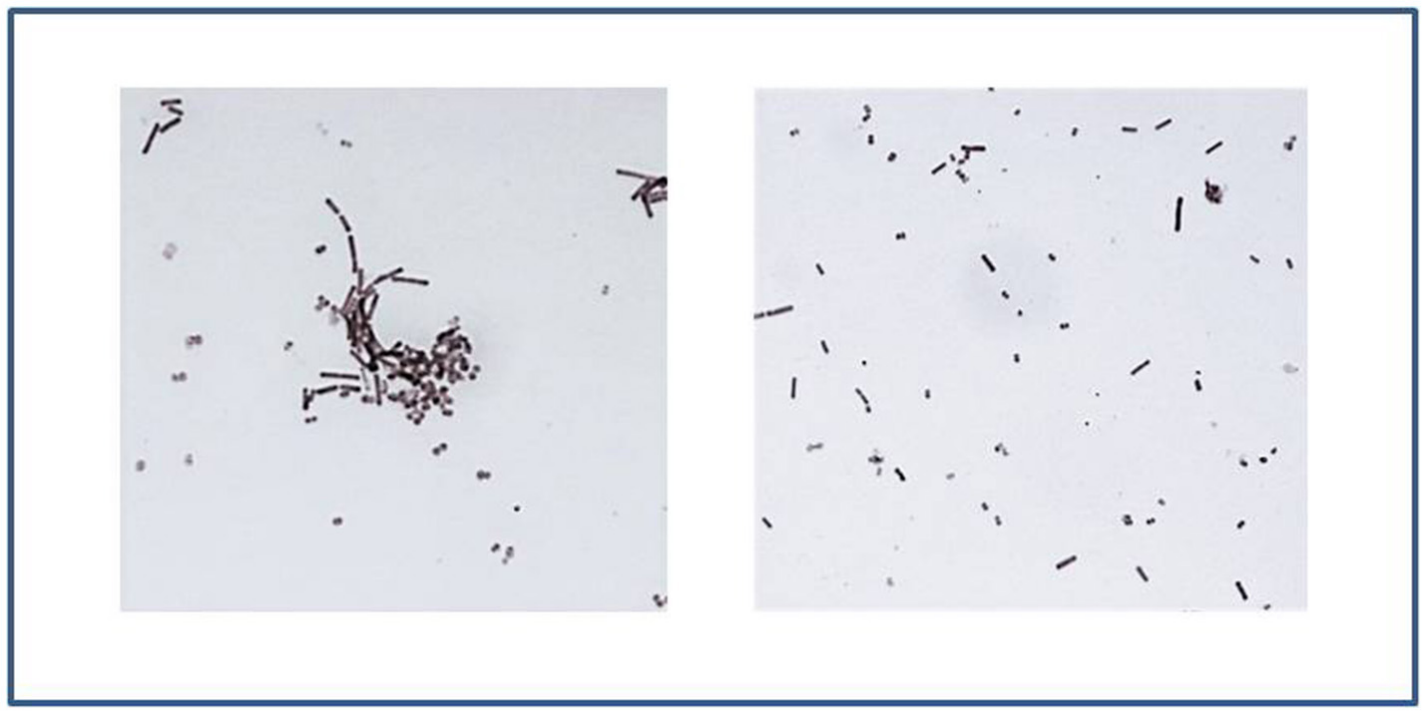
*Lactobacilli* interaction with piliated GC. On the left: *L. crispatus* BC1 shows high levels of interaction with GC after 7 min contact time, as underlined by the formation of lactobacilli-gonococci cells aggregates. On the right: after an interaction of 7 min, the ability of *L. crispatus* BC6 to aggregate with gonococci was excluded. Gram-stained images at 1,000 × magnification.

At short contact times, *L. crispatus* BC1 and BC3 showed the highest levels of co-aggregation with GC, indeed chains of lactobacilli were incorporated into GC cells. On the contrary, for the remaining *Lactobacillus* strains, the ability to aggregate was partially or totally excluded. Figure 5 shows some examples of lactobacilli-GC interaction after 7 min contact time.

After an interaction of 60 min, all the strains belonging to *L. crispatus* species, as well as some strains of the other species (*L. gasseri* BC9 and BC14, *L. vaginalis* BC17) were able to aggregate with piliated gonococci, often arranged in microcolonies. Figure S4 shows Gram-stained examples of the interaction between lactobacilli (BC1 and BC10) and GC, after 60 min contact time. It is worth to be underlined that the ability of BC1 to aggregate with gonococcal cells was retained even after repeated cycles of washes and centrifugation, thus corroborating the hypothesis of a specific bacterial interaction (Figure S5).

## Discussion

The emergence and spread of antimicrobial resistance in *N. gonorrhoeae*, together with the significant morbidity associated with untreated gonococcal infections in women, underline the need of new strategies for the control of gonorrhea (ECDC, 2016). In this context, the use of lactobacilli probiotic formulations could represent an intriguing new approach for the prevention of gonococcal infections.

Therefore, the aim of this study was to assess the antimicrobial activity of 14 vaginal *Lactobacillus* strains against GC, mimicking what happens in the first phases of the natural course of the gonococcal infection in the female genital tract.

Considering that the vagina is the port of entry for gonococci, the creation of a vaginal environment able to counteract GC viability could prevent its reaching and colonization to the endocervical epithelia, primary target of the infection (Spurbeck and Arvidson, 2010). The vaginal lactobacilli could act as a barrier against GC invasion by means of the creation of an “adverse” vaginal niche, through various mechanisms: (i) production of various antimicrobial compounds secreted in the vaginal fluids, (ii) co-aggregation capability, (iii) release of derived cell-free components (e.g., released surface components with biosurfactant activity).

In order to reproduce the interaction between lactobacilli and GC in the vaginal niche, we performed “inhibition” experiments, evaluating the ability of two different fractions of lactobacilli (cell pellets and cell-free supernatants) in reducing GC viability.

To this purpose, we tested *Lactobacillus* strains belonging to the most common species found in the vaginal microbiota of healthy reproductive-age women (Ravel et al., 2011). Despite its high incidence in the vaginal microbiota, we did not include any strain of *L. iners*, considering the technical issues in routine culturing (stringent nutritional requirements and very low oxygen tolerance) and its close correlation with vaginal dysbiosis (Macklaim et al., 2013).

At first, our results strengthen the importance and the role of acidification in the inhibition of GC by lactobacilli, in line with previous reports. Indeed, it has been demonstrated that gonococci grow well in a pH range comprised between 6.4 and 7.3 (Morin et al., 1980), whereas in case of lower pH values (4.8–5.0) a significant decrease in gonococcal survival occurs (Zheng et al., 1994). Moreover, it has been reported that the inhibition of GC by vaginal lactobacilli can be directly due to the acidic production as well as to the modulation of bioactive substances in an acidic milieu (St Amant et al., 2002; Graver and Wade, 2011).

Here, we showed that the acidic environment, produced by vaginal lactobacilli metabolism, is extremely crucial in counteracting GC growth and that lactobacilli activity against gonococci is pH- and time-dependent. Indeed, only when the pH values of lactobacilli supernatants were <4.0, a complete abolishment of GC viability was found, even at short contact times.

The experiments with organic/inorganic acid solutions confirmed the strict correlation between the pH levels and the anti-gonococcal activity. This finding is in line with what has been shown for *Chlamydia trachomatis* (Nardini et al., 2016). In this context, only for pH values < 4.0, no differences in the anti-GC effect were noticed between hydrochloric and lactic acid solutions. In case of higher pH levels, only lactate retained a good anti-gonococcal activity, indicating that the presence of H^+^ ions is necessary but not sufficient to kill gonococci.

It is known that lactate can be used by gonococci for energy production and synthesis of cell wall component precursors, as well as for the enhancement of LPS sialylation, affecting the pathogenicity *in vivo* (Yates et al., 2000; Smith et al., 2001, 2007). On the other hand, pH values can have a profound impact on the carbon utilization in GC, redirecting the biochemical pathways and changing the chemical composition of the gonococcal cells (Morse and Hebeler, 1978). We can thus hypothesize that, in the context of the present data, the fostering effect of lactate on gonococci growth is overcome by low pH values, leading to a GC viability reduction as predominant effect.

Even though a modulation of other anti-GC components by the lactobacilli acidification cannot be completely ruled out, we supposed that the inhibitory action of supernatants was primarily due to the acidic production rather than to other bioactive molecules. Such hypothesis was suggested, on one hand, by the complete absence of activity against GC in case of buffered supernatants, and, on the other, considering that, at equivalent pH values, CFS showed similar anti-GC effects compared to lactic acid solutions.

Overall, our data underline that acidification capabilities of individual lactobacilli are fundamental in the activity against GC and should be taken into account for probiotic strain selection.

When “inhibition” experiments were performed with lactobacilli cell pellets, we found that only a few strains, belonging to *L. crispatus* species, were able to reduce GC viability at short contact times and that the lactobacilli viability was crucial for the GC-killing effect.

We noticed that the ability of lactobacilli to interact and aggregate with gonococci was strictly correlated with their capacity in counteracting GC viability. In line with previous reports, these results highlight the importance of bacterial co-aggregation as an antimicrobial mechanism (Boris et al., 1997; Vielfort et al., 2008). But, if lactobacilli-gonococci co-aggregation was previously considered only for its ability in detaching gonococci from the epithelial cell surface (Vielfort et al., 2008), in this study, the bacterial interaction was considered as a direct killing mechanism.

In addition, we evaluated the biosurfactant capacity and the anti-gonococcal activity of lactobacilli released surface components with a molecular weight >10,000. In this context, RSC isolated from *L. crispatus* BC1 showed remarkable biosurfactant properties and GC killing effect, even at short contact times, in presence of a dose-dependent response. Since BC1 RSC was characterized by a pH value of 5.5, the anti-GC effects could be ascribed to various amphiphilic compounds (glycolipids, lipopeptides, polysaccharide-protein complexes, phospholipids, fatty acids), rather than to an acidic environment. Although other mechanisms cannot be ruled out, we suppose that, during the bacterial interaction, surface biosurfactant components, especially derived from “highly-aggregating” lactobacilli (e.g., BC1) are able to interfere with gonococci and reduce their viability. On the contrary, the cells of non-aggregating strains (e.g., BC10) were completely ineffective against GC, although in presence of a biosurfactant activity of RSC. To confirm this, the effects of lactobacilli biosurfactant components on the gonococcal external membrane should be assessed by means of additional studies.

In this context, the antimicrobial potential of biosurfactants isolated from lactobacilli against pathogens has been largely investigated (Satpute et al., 2016; Merghni et al., 2017). So far, the *in-vitro* anti-gonococcal activity has been demonstrated for some fatty acids and monoglycerides (Bergsson et al., 1999), but, no exhaustive data about the role of lactobacilli RSC in counteract GC viability is yet available.

Finally, it is worth mentioning that lactobacilli preserved completely their viability after the interaction with gonococci. This aspect is particularly significant, considering that, *in vivo*, lactobacilli can keep on their “health-promoting” activity in the vaginal niche.

Globally, we noticed that *L. crispatus* species, even in presence of a strain-specific activity, showed the best antimicrobial profile against GC compared to the other *Lactobacillus* species. The significant activity shown at short contact times (7 min) is of particular interest, considering that a such rapid GC killing could effectively prevent GC overcoming of the vaginal niche and its incoming to the endocervical epithelium.

The central role of *L. crispatus* in the maintenance of a healthy vaginal microbiota has been largely described. Indeed, *L. cripatus* has been previously indicated as one of the most active species against several urogenital pathogens, including GC (Breshears et al., 2015; Nunn et al., 2015; Parolin et al., 2015), suggesting that a vaginal microbiota dominated by this species can prevent different uro-genital and sexually transmitted microorganisms simultaneously, by means of a plethora of mechanisms. By virtue of its antimicrobial properties, attempts have been done to formulate *L. crispatus* in vaginal inserts for topical treatment of uro-genital infections (Vitali et al., 2016).

To our knowledge, this is the first report investigating the anti-GC activity of a wide panel of vaginal *Lactobacillus* strains, belonging to different species. We identified specific vaginal *Lactobacillus* strains (e.g., *L. crispatus* BC1 and BC3) able to interfere with the gonococcal infection by multiple strategies (GC killing due to low pH values, reduction of GC viability by cell-pellets) and we elucidated some of the mechanisms of action.

Additionally, this work can shed light on the use of lactobacilli probiotics as preventive approaches for gonococcal infections in women.

Further studies are needed for a thorough understanding of the molecular basis of the interaction between GC and lactobacilli and to evaluate the composition of released surface components with biosurfactant properties able to abolish GC viability.

## Author contributions

AM, BV, CF, and RC: conceived and designed the experiments; CF and MS: performed the experiments; CP and CF: analyzed the data; AM, BV, CP, and RC: contributed reagents, materials, analysis tools; AM, BV, CF, and CP: wrote the paper. All the authors read, reviewed, and approved the final manuscript.

### Conflict of interest statement

The authors declare that the research was conducted in the absence of any commercial or financial relationships that could be construed as a potential conflict of interest.
