# Supplementary material for: Vaginal Lactobacilli Reduce Neisseria gonorrhoeae Viability through Multiple Strategies: An in Vitro Study
Source: Front Cell Infect Microbiol. 2017 Dec 6;7:502. doi: 10.3389/fcimb.2017.00502 (PMC5723648; doi:10.3389/fcimb.2017.00502)

**Supplementary materials**

**Figure S1. Effect of lactic and hydrochloric acid buffered MRS medium on GC viability.** MRS broth buffered to different pH values (3.4, 3.7, 4.0, 4.4, 4.7, 5.0, 5.3) with lactic acid (LA) or hydrochloric acid (HCl) was used for ‘inhibition’ experiments against GC at two different time points (7 and 60 minutes; dotted and gray bars, respectively). GC viability was evaluated as number of gonococci/mL and expressed as % of gonococci survival. The results were expressed in percentage compared with control (K) (1 × 10^8^ gonococcal cells incubated in MRS broth, pH 6.0), taken as 100% (black bar). Bars represent mean values, whereas error bars represent SEM. Statistical significance was determined at *P* < 0.05 (*), *P* < 0.01 (**) and *P* < 0.0001 (***).

**
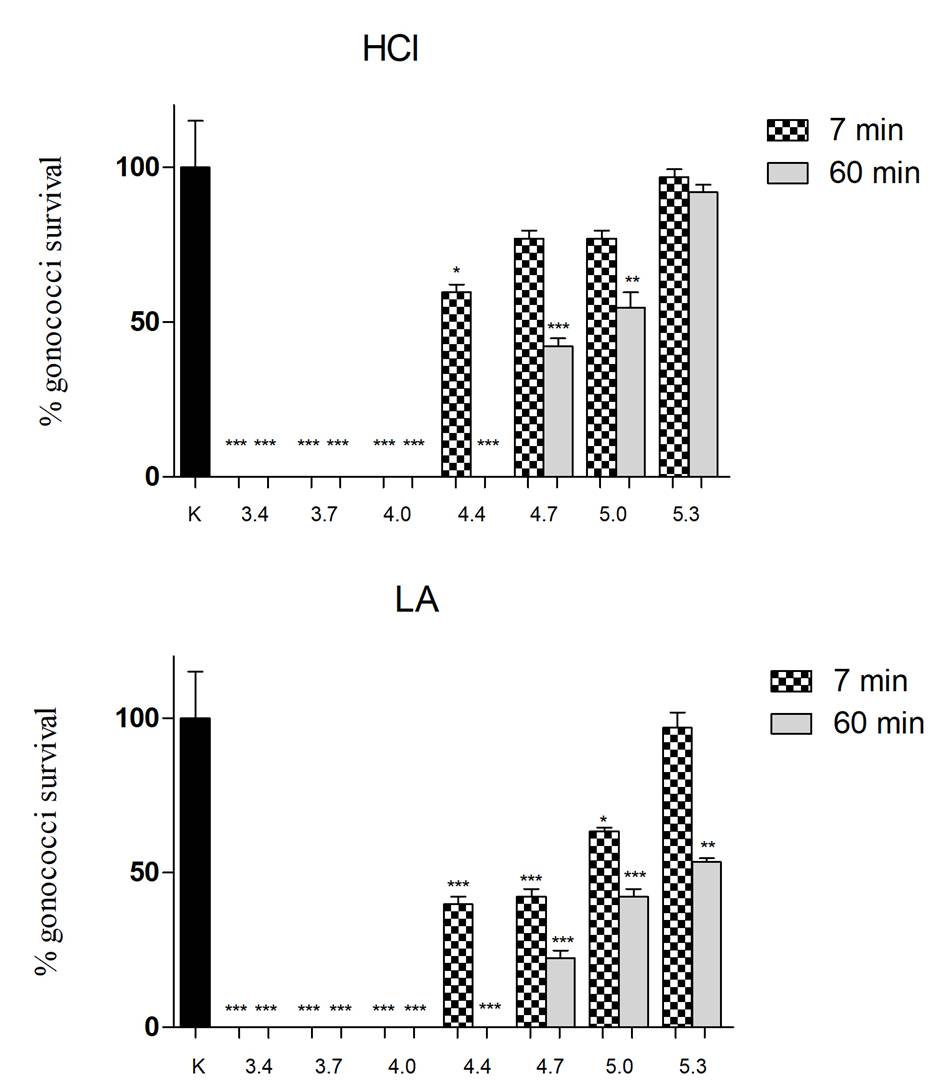
**

**
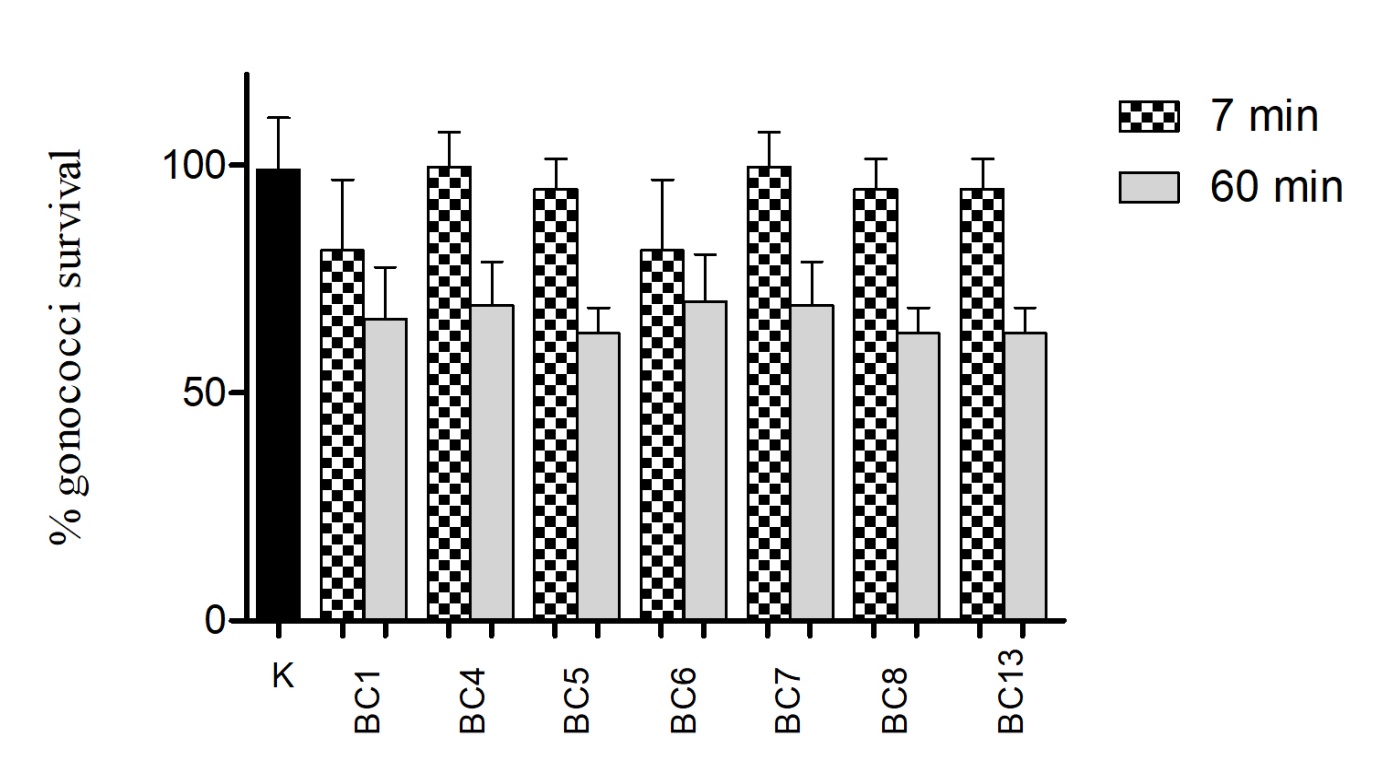
Figure S2. Effect of lactobacilli buffered supernatants on GC viability.** The supernatants of highly active *Lactobacillus* strains (BC1, BC4, BC5, BC6, BC7, BC8, BC13) were buffered to pH 6.0 and ‘inhibition’ experiments were performed. GC viability was evaluated as number of gonococci/mL and expressed as % of gonococci survival. The results were expressed in percentage compared with control (K) (1 × 10^8^ gonococcal cells incubated in MRS), taken as 100% (black bar). Bars represent mean values, whereas error bars represent SEM. Statistical significance was determined at *P* < 0.05 (*).

**Figure S3. ‘Inhibition’ experiments with methanol and proteinase K-treated lactobacilli.** Cell pellets of *L.* *crispatus* strains BC1 and BC3 were treated with methanol (metOH) and proteinase K (ProK), prior to the ‘inhibition’ experiments against GC. GC viability was evaluated as number of gonococci/mL and expressed as % of gonococci survival. The results were expressed in percentage compared with control (K) (1 × 10^8^ gonococcal cells incubated in PBS), taken as 100% (black bar). Bars represent mean values, whereas error bars represent SEM. Statistical significance was determined at *P* < 0.05 (*).

**
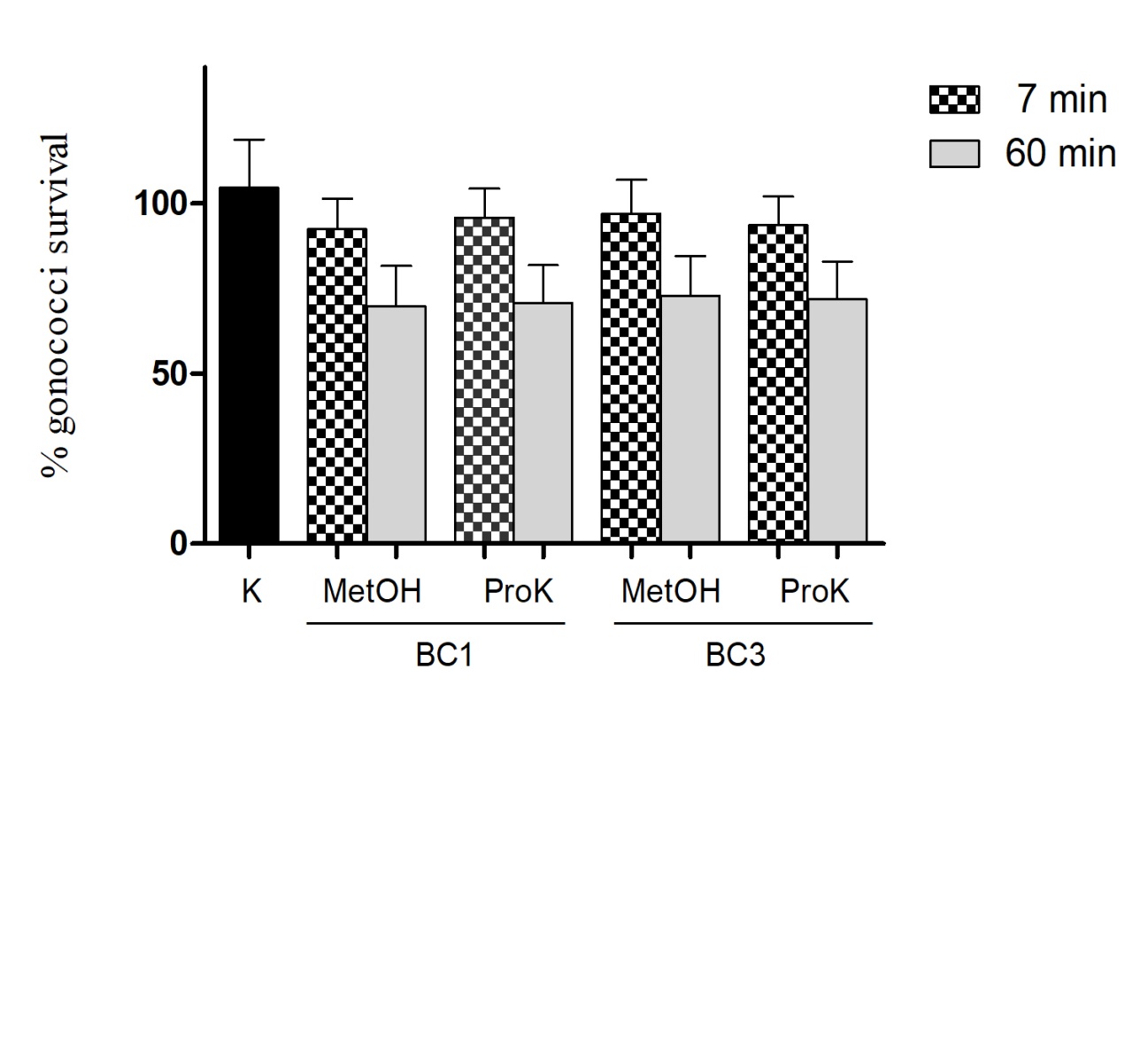
**

**Figure S4. Gram-stained examples of lactobacilli-GC interaction after 60 minutes**.

**(A)** *L. crispatus* BC1 shows a significant ability to interact and aggregate with gonococcal cells. **(B)** Interaction between GC and *L. gasseri* BC10, a strain with low aggregation capability. (1000× magnification).

**
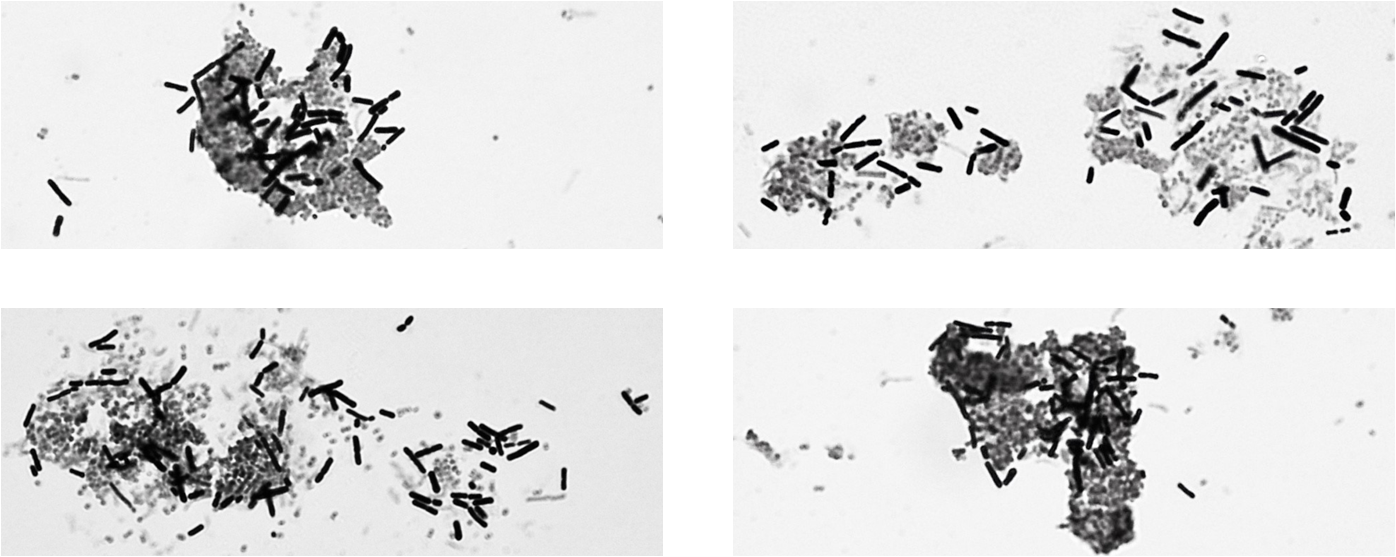
(A)**

**(B)**

**
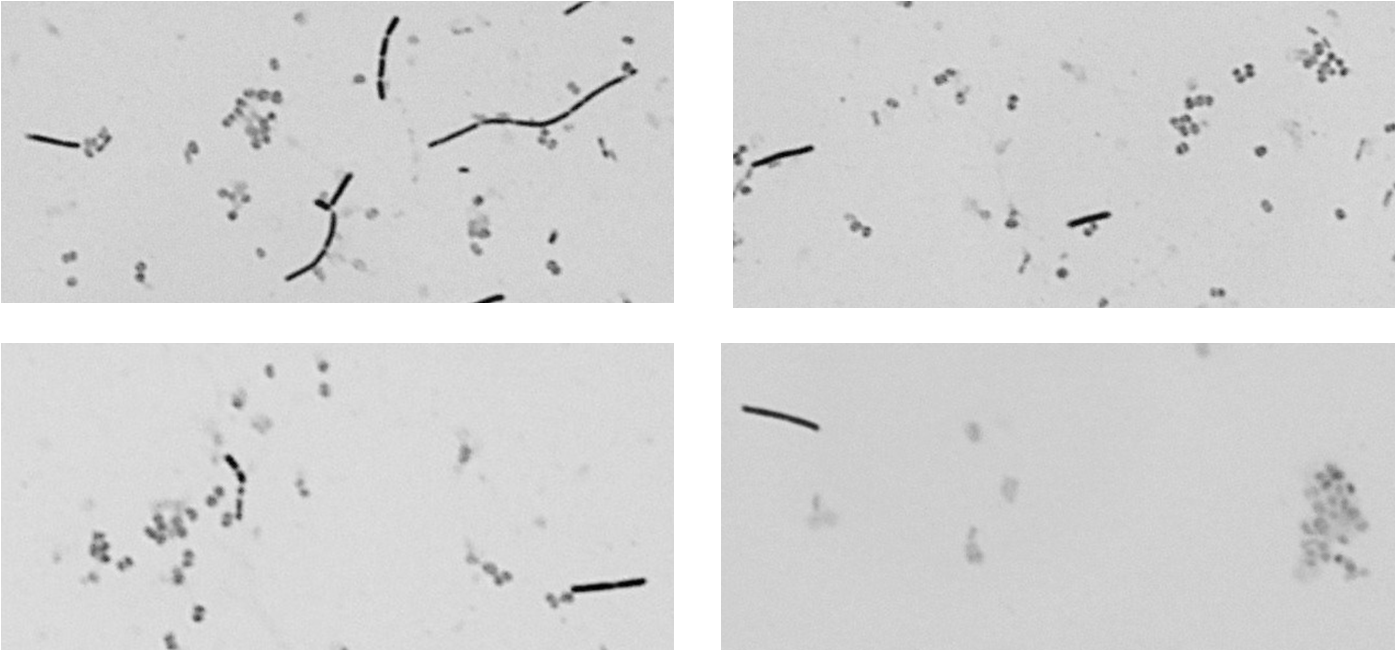
**

**Figure S5. *L. crispatus* BC1-GC interaction after repeated cycles of washes and centrifugation.** Gram-stained examples of BC1-GC aggregation, 1000× magnification.


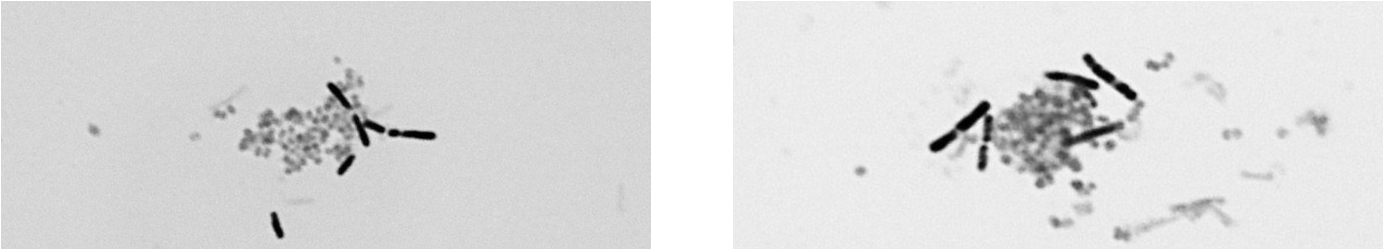

Supplement: Supplementary file 1 [file DataSheet1.docx]
